# β-Sitosterol Inhibits Rheumatoid Synovial Angiogenesis Through Suppressing VEGF Signaling Pathway

**DOI:** 10.3389/fphar.2021.816477

**Published:** 2022-02-28

**Authors:** Kai Qian, Xue-Xia Zheng, Chen Wang, Wen-Guang Huang, Xiao-Bao Liu, Shu-Di Xu, Dan-Kai Liu, Min-Ying Liu, Chang-Song Lin

**Affiliations:** ^1^ Guangzhou University of Chinese Medicine, Guangzhou, China; ^2^ Postdoctoral Research Station, Guangzhou University of Traditional Chinese Medicine, Guangzhou, China; ^3^ Department of Rheumatology, First Affiliated Hospital of Guangzhou University of Chinese Medicine, Guangzhou, China

**Keywords:** rheumatoid arthritis, *β-*sitosterol, VEGFR2, angiogenesis, endothelial cells, collagen-induced arthritis

## Abstract

**Background:** Rheumatoid arthritis (RA) is a chronic disabling inflammatory disease that causes synovial angiogenesis in an invasive manner and leads to joint destruction. Currently available pharmacotherapy for RA has unwanted side effects and limitations. Although anti-angiogenic therapy is regarded as a new potential treatment for RA, only a few anti-angiogenic drugs are available. An increasing number of studies have shown that *β*-sitosterol (BSS) may exert inhibitory effects against angiogenesis. However, the mechanisms involved are still unclear.

**Methods:** Based on the results of the gene set enrichment analysis (GSEA) of the transcriptome data of endothelial cells from RA patients, we evaluated the pharmacological effects of BSS on the tube formation, cell proliferation, and migration of human umbilical vein endothelial cells (HUVECs). Furthermore, the effects of BSS treatment on vascular endothelial growth factor receptor 2 (VEGFR2) were determined using molecular docking and Western blotting. Additionally, in the presence or absence of BSS, synovial angiogenesis and joint destruction of the ankle were investigated in collagen-induced arthritis (CIA) mice. The effect of BSS treatment on VEGFR2/p-VEGFR2 expression was verified through immunohistochemical staining.

**Results:** The immunohistochemistry results revealed that BSS treatment inhibited angiogenesis both *in vitro* and *in vivo.* In addition, the results of 5-ethynyl-2′-deoxyuridine and cell cycle analysis showed that BSS treatment suppressed the proliferation of HUVECs, while the Transwell migration and stress fiber assays demonstrated that BSS treatment inhibited the migration of HUVECs. Notably, the inhibitory effect of BSS treatment on VEGFR2/p-VEGFR2 was similar to that of axitinib. In CIA mice, BSS also exerted therapeutic effects on the ankles by reducing the degree of swelling, ameliorating bone and cartilage damage, preventing synovial angiogenesis, and inhibiting VEGFR2 and *p*-VEGFR2 expression.

**Conclusion:** Therefore, our findings demonstrate that BSS exerts an inhibitory effect on synovial angiogenesis by suppressing the proliferation and migration of endothelial cells, thereby alleviating joint swelling and bone destruction in CIA mice. Furthermore, the underlying therapeutic mechanisms may involve the inhibition of VEGF signaling pathway activation.

## Introduction

Rheumatoid arthritis (RA) is a common inflammatory disease with a prevalence of about 1% of people worldwide (O’neil et al., 2020), leading to joint deformity and disability constituting a burden both on individuals and society. RA is characterized by persistent synovitis and developing synovial angiogenesis, which leads to subsequent hyperplasia of the synovium and the erosion of cartilage and bone (Mcinnes and Schett, 2011; Bottini and Firestein, 2013). In the course of RA, active tissue neovascularization is necessary for the expansion, hyperplasia, and invasiveness of synovium (Fearon et al., 2016). Accordingly, RA was as well regarded as one of the “angiogenic family of diseases” because of the notably increased number and density of new synovial blood vessels in synovium. What is more, increasing evidence support that angiogenesis appears to be a central part involved in the development and maintenance of RA (Leblond et al., 2017).

In the progress of angiogenesis, endothelial cells (ECs) are an indispensable source of new synovial blood vessels (Leblond et al., 2017). Vascular endothelial growth factor (VEGF) is a main “on” switch controlling nearly all steps of angiogenesis (Apte et al., 2019), and vascular endothelial growth factor receptor 2 (VEGFR2) is the principal endothelial VEGF signaling receptor (Simons et al., 2016). VEGF binds to homologous VEGFR2 and induces phosphorylation of Y1173 and Y949 sites in VEGFR2. Phosphorylated Y1173 binds and activates phosphorylated phospholipase C *γ* (PLCγ), while PLCγ mediates the activation of the ERK1/2 pathway, leading to gene transcription changes that affect biological processes such as cell migration, proliferation, and homeostasis (Sakurai et al., 2005). The phosphorylation of Y949 leads to the activation of sarcoma gene (c-SRC) at the cell-to-cell junction, which determines the downstream signaling events of cell shape, survival, and vascular permeability (Li et al., 2016). Nowadays, anti-VEGF/VEGFR2 therapies seem to be beneficial for animal models of RA and also have a great therapeutic promise for RA (Le and Kwon, 2021), such as Avastin (Bevacizumab, a VEGF humanized monoclonal antibody) (Wang et al., 2013) and Sorafenib (a proangiogenic receptor tyrosine kinase inhibitor, which mainly suppresses the expression of VEGFR2) (Wang et al., 2018).

With a long history of nutritional complement and pharmaceutical products, BSS is a common bioactive phytosterol that can be derived from many kinds of plants. It has antioxidant, cholesterol-lowering, anti-inflammatory effects, and is widely used in pharmaceutical raw materials and functional foods (Babu and Jayaraman, 2020). Phytosterols were reported to be involved in various mechanisms, such as inhibiting cancer cell growth, angiogenesis, and promoting cancer cells apoptosis (Woyengo et al., 2009). In addition, the expression of VEGF was decreased by BSS in renal tissue, and angiogenesis was inhibited by BSS in renal cell carcinoma (Sharmila and Sindhu, 2017). As we know, the characteristics of synovial angiogenesis were similar to tumor angiogenesis (Fearon et al., 2016). Therefore, we hypothesized that BSS may inhibit synovial angiogenesis in RA by affecting ECs, thereby reducing joint swelling and joint destruction. In our study, we implemented the gene set enrichment analysis (GSEA) method to analyze the transcriptome of synovial ECs in RA, and results showed that ECs were significantly enriched in proliferation and migration. Furthermore, we found that VEGF, PI3K-AKT-mTOR, and TGF-β signaling pathways, related to angiogenesis, were activated in ECs. Therefore, we used human umbilical vein endothelial cells (HUVECs) and collagen-induced arthritis (CIA) mice to explore the effect of BSS on synovial angiogenesis and its possible mechanism.

## Materials and Methods

### Reagents and Antibodies

BSS (drug purity ≥95%, batch number S1270) was derived from Sigma-Aldrich (St. Louis, MO, United States). For *in vivo* experiments, BSS was dissolved in anhydrous ethanol to 200 mM and stored at −20°C, and then diluted with EC medium (ECM). For *in vitro* experiments, BSS was dissolved in corn oil using anhydrous ethanol as cosolvent (final concentration of ethanol was 5%). Axitinib (drug purity was 99.76%, lot number S1005) was derived from Selleck (Houston, TX, United States). Recombinant human TNF-α (lot number 300-01A) and Recombinant human VEGF_165_ (lot number 100–20) were purchased from Peprotech (Cranbury, NJ, United States). ECM (lot number 1001) was purchased from ScienCell (Carlsbad, CA, United States). In addition, anti-VEGFR2 (lot number AF6281), anti-phospho-VEGFR2 (lot number AF3279) antibodies were obtained from Affinity Biosciences LTD. (Changzhou, Jiangsu, China). Anti-phospho-AKT (lot number 9271S) and anti-phospho-smad2 (lot number 3108S) antibodies were obtained from CST (Danvers, MA, United States).

### Animal Experimental Design

Twenty-four male DBA/1 mice (7–8 weeks), weighing 18 ± 2 g, were purchased from Beijing Weitong Lihua Laboratory Animal Technology, license number SCXK (Beijing) 2016-0011, animal certificate number 1100112011028365. The animals (animal ethics number 2020006) were maintained in the Experimental Animal Centre of the First Affiliated Hospital of Guangzhou University of Traditional Chinese Medicine, and experimental unit license number: SYXK [Guangdong] 2018-0092. This study was reviewed and approved by the Ethics Committee of the First Affiliated Hospital of Guangzhou University of Traditional Chinese Medicine, and conducted therapeutic intervention on laboratory animals in accordance with the NIH Laboratory Animal Care and Use Guidelines.

CIA mice were guided by a previous study (Pan et al., 2018), and the mice were randomly divided into four experimental groups (*n* = 6 each): normal control + vehicle (NC group), CIA + vehicle (CIA group), CIA + methotrexate (MTX group), and CIA + BSS (BSS group). The mice were treated *via* gastric irrigation of either vehicle, MTX (1 mg/kg every 3 days) or BSS (100 mg/kg daily) for 28 days, beginning on the day after the second immunization. Arthritis index score: scoring was initiated on day 21 and subsequently performed every 3 days (Li et al., 2017).

### Cell Culture

The HUVECs purchased from ScienCell (lot number 28433) were identified by CD31 fluorescent staining. The HUVECs were cultured in ECM, at 37 °C and 5% CO_2_. Operations such as medium exchange, subculture, cryopreservation, resuscitation, or seeding were performed according to the state and density of the cells. In this study, 4–6 generations of cell cultures were tested.

### Cell Counting Kit-8 Assay

The HUVECs were inoculated (6 × 10^3^ cells/well) in 96-well plates and then treated with different dosage groups of BSS for 24 h, each group included five duplicate wells, and the experiment was repeated thrice. After discarding the culture medium, the plates were washed with PBS, and 100 µl of fresh complete medium and 10 µl CCK-8 assay reagent (Dojindo, Kunamoto, Japan) were added to each well, and the cells were cultured for another 1 h. Absorbance (A value) was measured at 450 nm using a microplate reader, and cell viability was subsequently assessed.

### Tube Formation Assay

The Normal control + vehicle (NC group), VEGF + vehicle (VEGF group), and VEGF + BSS groups were first established. A 100-μl cell suspension (4 × 10^5^ cells/ml) was placed into each well of a 96-well plate, coated with 35 μl/well matrix adhesive (BD Biosciences, CA, United States). The HUVECs were treated with vehicle or BSS (10 and 20 μM) and stimulated with or without VEGF (50 ng/ml) for 6 h. The tube formation of HUVECs was observed and photographed using an inverted phase contrast microscope (Olympus, Tokyo, Japan). The Angiogenesis Analyser plug-in in ImageJ software (NIH, Bethesda, MD, United States; http://rsb.info.nih.gov/nih-image/) was used to analyze the number of tube-cavity nodes and connection intersections (Carpentier et al., 2020).

### Microarray Data Analysis

Following the experimental design, the gene expression profile data of ECs from 18 RA patients and 11 healthy controls in the GSE121894 dataset under basic conditions were selected (Leblond et al., 2020). GSEA was performed through the “clusterProfiler” R package, with “c5.all.v7.4.symbols.gmt” and “c2.cp.kegg.v7.4.symbols.gmt” as the reference gene set.

### Proliferation Assays

The HUVECs (6 × 10^3^ cells/well) were treated with vehicle or BSS (10 and 20 μM) and stimulated with or without VEGF (50 ng/ml) for 24 h. Cell proliferation was assessed using Apollo 488 Cell Proliferation Kit (Ruibo Biotechnology Co., Ltd., Guangzhou, China). Image was obtained using an inverted fluorescence microscope (Olympus) and ImageJ software (NIH) was used to count the 5-ethynyl-2′-deoxyuridine positive (EdU) cells and Hoechst^+^ cells in the photomicrographs. The positive rate of EdU staining was calculated using the formula: (positive rate = EdU^+^ cell number/Hoechst^+^ cell number).

### Cell Cycle Assays

The HUVECs (3 × 10^5^ cells/well) were treated with vehicle or BSS (10 and 20 μM) and stimulated with or without VEGF (50 ng/ml) for 24 h. The samples were centrifuged at 1,000 × *g* for 5 min to collect cells, and the supernatant was discarded. The cells were washed twice with cold PBS, and the precipitate was fixed with pre-cooled 75% ethanol overnight at 4°C. The fixed cells were collected by centrifugation at 1,000 × *g* and the supernatant was discarded. Then, 1 ml of PBS was added, and the cells were incubated for 15 min at 20–25°C. The cells were collected again by centrifugation and the supernatant was discarded. A 500-µl propidium iodide staining solution (Beyotime Biotechnology, Shanghai, China) was added to each tube, and the cells were incubated for 30 min. Accuri™ C6 flow cytometer (BD Biosciences) and FlowJo software (BD Biosciences) were used for cell cycle detection and analysis, respectively.

### Transwell Migration Assays

A 100-µl cell suspension (4 × 10^5^ cells/ml) was added to the upper chamber (total volume: 200 μl, with serum-free medium as solvent) of the Transwell apparatus (Costar, NY, United States), while medium (600 µl) containing 5% FBS was added to the lower chamber. The HUVECs were treated with vehicle or BSS (10 and 20 μM) and stimulated with or without VEGF (50 ng/ml) for 24 h. The migrated cells were stained with 0.1% crystal violet (Beyotime Biotechnology). Cells that migrated to the lower chamber were photographed under an inverted phase contrast microscope (Olympus). ImageJ software (NIH) was used to count the cells, and the average number of cells were determined.

### Stress Fiber Fluorescent Staining

The HUVECs (2 × 10^4^ cells/well) were seeded in 24-well plates, treated with vehicle or BSS (10 and 20 μM), and stimulated with or without tumor necrosis factor-alpha (TNF-α, 20 ng/ml) for 24 h. Then, the HUVECs were fixed on a glass slide with 4% paraformaldehyde for 20 min and infiltrated with 0.1% Triton X-100 in PBS for 5 min at 37°C. The cells were blocked with 1% BSA for 20 min, and incubated with Invitrogen Alexa Fluo™ r-546 phalloidin (Thermo Fisher Scientific, Waltham, MA, United States) for 20 min at 37°C to stain the F-actin. Subsequently, the glass cover was fixed on the glass slide using DAPI anti-fading mounting medium (Beyotime Biotechnology). Images were obtained using a laser confocal microscope (×630 oil objective). ImageJ software (NIH) was used for fluorescence quantification (Jensen, 2013), to obtain the arbitrary unit (AU). The mean was computed, and the experiment was repeated thrice.

### Molecular Docking

For molecular docking, we used CB-Dock (http://cao.labshare.cn/cb-dock/), which employs a new curvature-based cavity detection method to predict the binding sites of specific proteins, calculate their centers and sizes, and integrate them with AutoDock Vina, with an optimization success rate that reaches 70% (Liu et al., 2020). The VEGFR2 protein PDB format (PDB ID: 6GQQ) file was obtained from the Protein Database (http://www.rcsb.org). The ligand files in SDF format for BSS and axitinib compounds were obtained from PubChem (https://pubchem.ncbi.nlm.nih.gov/).

### Western Blot Analysis

The HUVECs were homogenized in a lysis buffer, and centrifugated at 12,000 × *g* for 15 min at 4°C. The protein concentration in the supernatant was determined using the BCA protein assay kit (Beyotime Biotechnology). Equal amounts of protein were separated using 7.5% or 10% PAGE Gel Rapid Preparation Kit (Yazyme Biotechnology, Shanghai, China) under denaturing and non-reducing conditions and then transferred to a PVDF membrane. The membrane was blocked with Protein Free Rapid Blocking buffer (Yazyme) for 1 h and incubated with the primary antibody overnight at 4°C. After washing thrice with TBST, the blots were incubated with the horseradish-coupled secondary antibody. The signals were visualized using enhanced chemiluminescence reagent (Pierce Biotechnology, Rockford, IL, United States) and recorded using ChemiScope 3500 mini chemiluminescence imaging system (Clinx, Shanghai, China).

### Histological Evaluation

The ankles were fixed after the mice were sacrificed. Decalcification and paraffin embedding were subsequently performed. Sections of the ankle samples (4 μm thick) were stained with hematoxylin and eosin (H and E) and safranin O-fast green (Sigma-Aldrich). A digital pathological scanner (3DHISTECH Ltd., Budapest, Hungary) was used for slide scanning.

### Micro-CT Scan

The ankle was fixed with paraformaldehyde, and the skeletal structure was scanned by micro-CT system (SKYSCAN 1172, BRUKER, Belgium). Scanning parameter: resolution 4 μm, voltage 80 kV, current 88 μA. Three-dimensional reconstruction was performed using CT-Vox software after scanning. The bone destruction score was analyzed by Micro-CT (the erosion degree of knee and ankle was divided into 0–4 points according to the median, medial, and lateral planes, and the total score was 12 points) (Najm et al., 2020), and the results were expressed as mean ± standard deviation.

### Immunohistochemistry Staining

Sections of the ankle samples (4 μm thick) were stained. After dewaxing, sections were submitted to antigen retrieval and further blocked with blocking fluid. The slices were rinsed in PBS for 5 min, 3 times, and then incubated with primary antibody (1:100 dilution) at 4°C overnight. After washing with PBS, the slices were incubated with secondary antibody for 1 h. Digital pathological scanner (3DHISTECH) was used for slide scanning, and three visual fields were selected at the joint site. ImageJ software was used to calculate the integral absorbance (IOD)/mm^2^ (Van Kuijk et al., 2010), and the mean was taken for statistical analysis.

### Statistical Analysis

The data were analyzed using SPSS software version 22.0 (IBM SPSS, Armonk, NY, United States) and GraphPad Prism version 5.0 (GraphPad, San Diego, CA, United States). Data were expressed as means ± standard deviations. The *t*-test, one-way analysis of variance (ANOVA), and Kruskal–Wallis non-parametric test were performed to evaluate the differences between experimental groups. The statistical significance was set at *p <* 0.05.

## Results

### BSS Treatment Inhibits the VEGF-Induced Tube Formation of HUVECs

**FIGURE 1:**
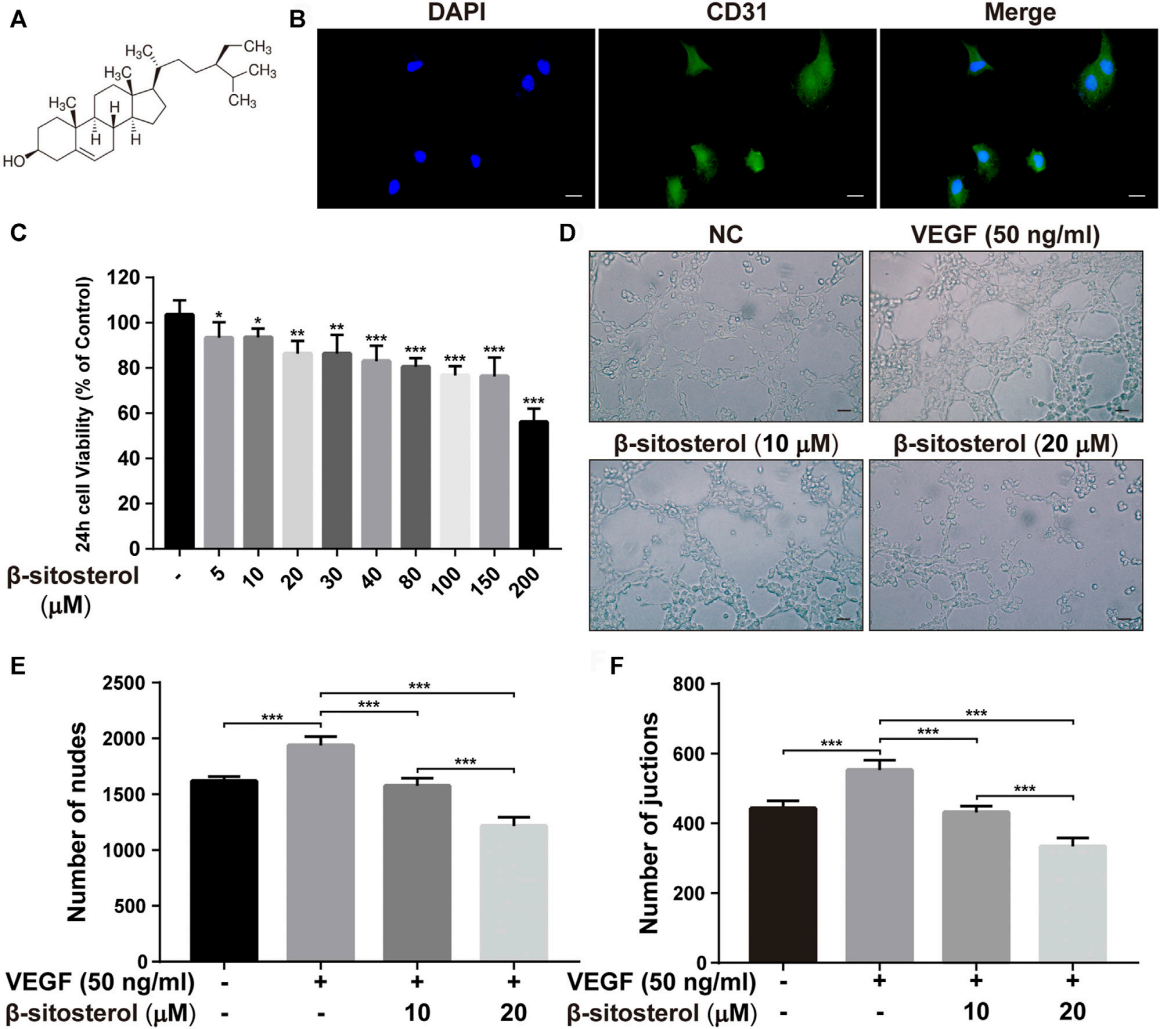
BSS inhibits angiogenesis in HUVECs. **(A)** BSS chemical formula (quoted from sigma official website). **(B)** CD31 fluorescent staining identification (×630, scale bar = 20 μm). **(C)** Effects of different concentrations of BSS on CCK-8 in HUVECs after 24 h (*n* = 3). **(D)** Representative images of tube formation at 6 h in HUVECs (×200, scale bar = 50 μm), *Y*-axis shows the nodes **(E)** and junction numbers **(F)** (*n* = 3). All data are shown as the mean ± SEM. *p < 0.05, **p < 0.01, ***p < 0.001.

The chemical formula of BSS is shown in Figure 1A. The HUVECs were successfully identified using CD31 fluorescent staining **(** Figure 1B **)**. The CCK-8 assay was used to evaluate the effects of BSS on HUVECs viability **(** Figure 1C **)**, Specifically, a cell viability of >80% was observed in cells treated with 10 and 20 μM BSS. Hence, these were identified as the non-toxic dosages and used for subsequent experiments.

To investigate the anti-angiogenic effect of BSS, we performed a tube formation test. We determined that BSS significantly inhibited VEGF-induced tube formation in HUVECs **(** Figure 1D **)**. Our data also showed that the two dosage groups of BSS significantly reduced the number of junction points **(** Figure 1E **)** and cross-junction points **(** Figure 1F **)** in the tube formation of HUVECs (*p* < 0.001).

### BSS Treatment Suppresses VEGF-Induced Proliferation of HUVECs

**FIGURE 2:**
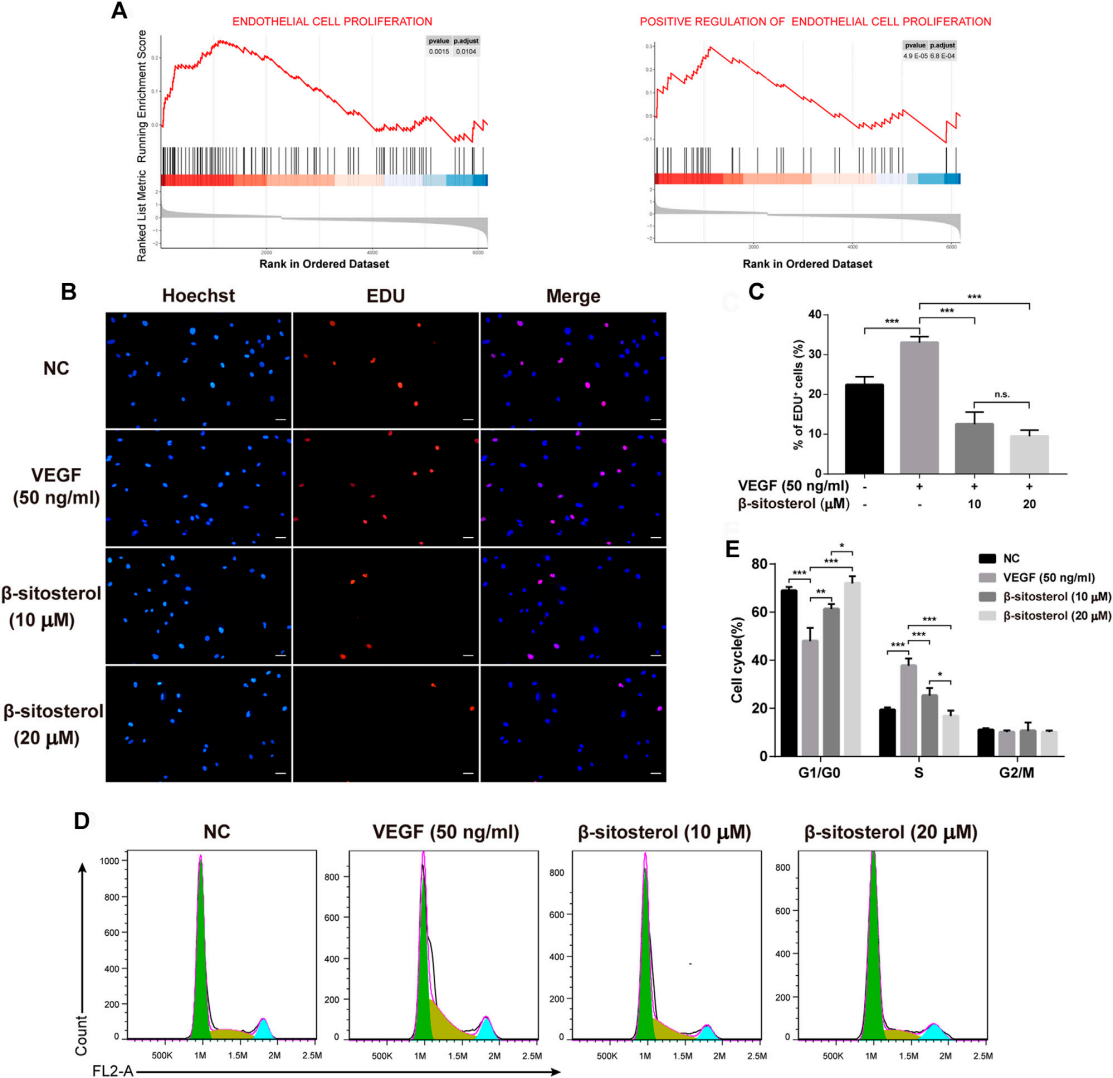
Inhibitory effect of BSS on HUVECs proliferation induced by VEGF. **(A)** GSEA analysis of ECs proliferation in the GSE121894 dataset. **(B)** Effect of BSS on proliferation of HUVECs (VEGF 20 ng/ml for 24 h) and analysis of proliferation rate **(C)** by EdU (×200, scale bar = 50 μm) (*n* = 3). **(D**) Effect of BSS on cell cycle of HUVECs and analysis of cell cycle data **(E**) by flow cytometry (*n* = 3). All data are shown as the mean ± SEM. *p < 0.05, **p < 0.01, ***p < 0.001, n.s. = not significant.

Angiogenesis is closely related to EC proliferation, and GSEA analysis of the GSE121894 dataset showed that “endothelial cell proliferation” and “positive regulation of endothelial cell proliferation” were significantly enriched in patients with RA, so we investigated the effect of BSS on endothelial cell proliferation **(** Figure 2A **)**. The EC transcriptome profile data were standardized and displayed (Supplementary Figure S1A), and the differential gene expression was displayed as a volcano graph (Supplementary Figure S1B).

To evaluate the inhibitory effect of BSS on the proliferation of HUVECs, we conducted EDU proliferation and cell cycle experiments. We determined that BSS significantly inhibited the VEGF-induced proliferation of HUVECs **(** Figures 2B,C **)**. Furthermore, cell cycle **(** Figure 2D **)** analysis showed that BSS induced S phase arrest in VEGF-stimulated HUVECs **(** Figure 2E **)**. These data suggest the inhibitory effect of BSS on cell proliferation of HUVECs.

### BSS Treatment Prevents VEGF and TNF-α Induced Migration of HUVECs

**FIGURE 3:**
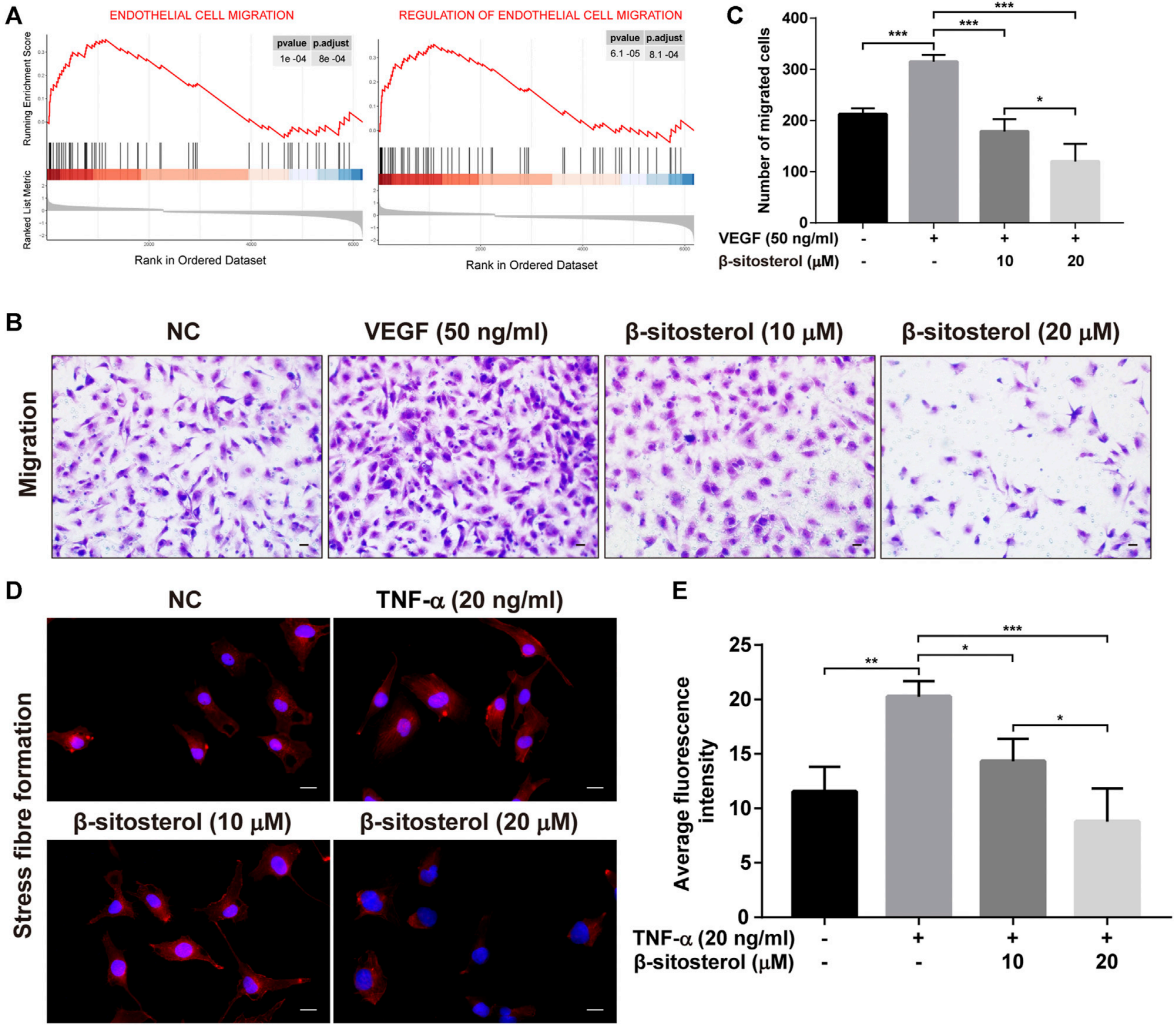
Inhibitory effect of BSS on HUVECs migration induced by VEGF or TNF-α. **(A)** GSEA analysis of ECs migration in GSE121894 dataset. **(B)** Representative images of cell migration in a modified Boyden chamber following HUVECs induced by VEGF stimulation (×200, scale bar = 50 μm). **(C)** *Y*-axis shows the number of migrated cells (*n* = 3). **(D)** Representative images of stress fiber formation on TNF-α stimulation (20 ng/ml for 24 h) (×630, scale bar = 20 μm), Nuclei are stained with DAPI (blue). **(E)** *Y*-axis shows fluorescence intensity quantified by ImageJ (*n* = 3). All data are shown as the mean ± SEM. *p < 0.05, **p < 0.01, ***p < 0.001.

It is well known that endothelial angiogenesis is closely related to endothelial cell migration, and GSEA analysis of GSE121894 dataset showed that “endothelial cell migration” and “regulation of endothelial cell migration” were significantly enriched in patients with RA. Therefore, we evaluated the effect of BSS on endothelial cell migration, measured using transwell assays **(** Figure 3A **)**. We determined that BSS treatment reduced VEGF-induced HUVECs migration **(** Figures 3B,C **)**. Since stress fibers provide the mechanical basis for cell migration (Fischer et al., 2021), we evaluated the effect of BSS on intensity of F-actin stress fibers. As shown in Figures 3D,E, BSS reduced the TNF-α-induced formation of stress fibers, further confirming the role of BSS in regulating EC migration. Collectively, our data indicate that BSS suppressed angiogenesis through, at least in part, regulating ECs migration.

### BSS Treatment Inhibits the TNF-α-Induced Expression of VEGFR2 and *p*-VEGFR2 in HUVECs

**FIGURE 4:**
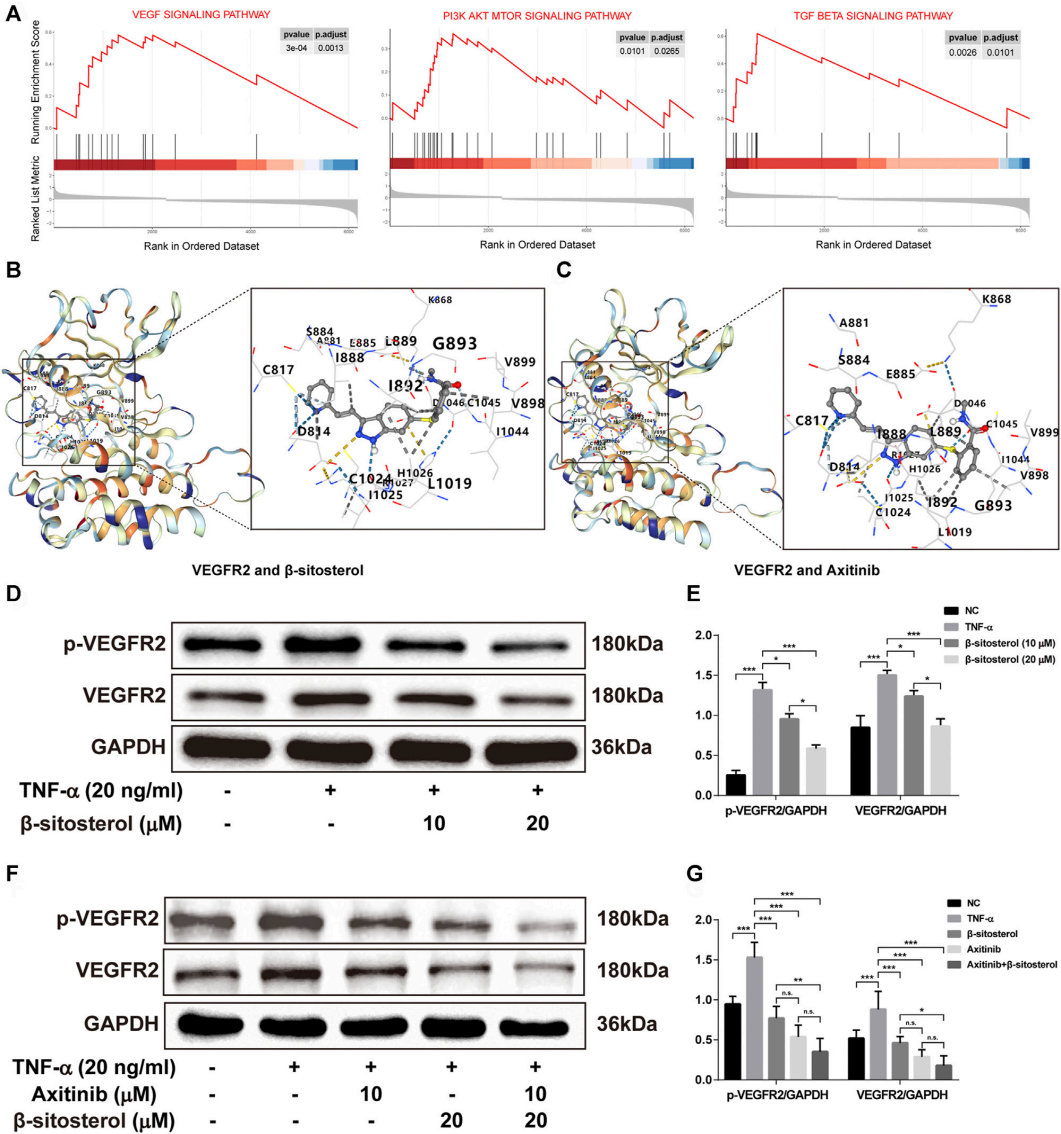
The effect of VEGF signaling pathway in TNF-α-induced HUVECs regulated by BSS. **(A)** GSEA analysis of KEGG signaling pathway in the GSE121894 dataset. The structural interactions between VEGFR2 and BSS **(B)** and between VEGFR2 and axitinib **(C)** were described by molecular simulation, and binding affinity of VEGFR2 with BSS and axitinib is shown in Table 1. **(D)** *p*-VEGFR2 and VEGFR2 protein levels in HUVECs (on TNF-α 20 ng/ml for 1 h) intervened with BSS dosage groups were detected by immunoblotting and the relative expression levels **(E)** of proteins were corrected by GAPDH (*n* = 3). (**F)** *p*-VEGFR2 and VEGFR2 protein levels in HUVECs intervened with BSS and axitinib were detected by immunoblotting, and the relative expression levels **(G)** of proteins were corrected by GAPDH (*n* = 3). All data are shown as the mean ± SEM. *p < 0.05, **p < 0.01, ***p < 0.001, n.s. = not significant.

GSEA was used to analyze the enrichment of ECs and angiogenesis-related signaling pathways in patients with RA. The results showed that VEGF signaling, PI3K-AKT-mTOR signaling pathway, and TGF-β signaling pathway were significantly activated in ECs of patients with RA **(** Figure 4A **)**. In addition, previous studies have shown that BSS inhibits activation of PI3K-AKT and TGF-β signaling pathways (Xu et al., 2018; Park et al., 2019); the AKT and Smad2 as the major proteins in these pathways were suppressed by BSS in phosphorylated protein level in our data (Supplementary Figure S2). However, the effect of BSS on VEGF signaling pathway is unknown.

To evaluate the binding ability of BSS with VEGFR2 by molecular docking technology, we selected axitinib as a positive control drug for comparison. The more negative the Vina score (kcal/mol) and the larger the binding cavity, the more stable the binding of the receptor to the ligand (Liu et al., 2020). The results showed that the Vina scores of BSS **(** Figure 4B **)** and axitinib **(** Figure 4C **)** with VEGFR2 were close (−9.0 vs. −8.9, respectively), and the binding cavities of BSS and axitinib to VEGFR2 were 1,633 **(** Table 1 **)**. With further verification by Western blot, the results showed that BSS (10 and 20 μM) significantly inhibited the expression of VEGFR2 and *p*-VEGFR2 **(** Figures 4D,E **)**. We also demonstrated that the inhibitory effect was more obvious under the combined treatment with axitinib and BSS **(** Figures 4F,G **)**.

**TABLE 1:** Binding affinity between VEGFR2 and BSS.

Compound name	Molecular formula	PubChem Cid	Molecular weight (g/mol)	Cavity size	Affinity (kcal/mol)
BSS	C_29_H_50_O	521199	414.7	1633	−9.0
Axitinib	C_22_H_18_N_4_OS	6450551	386.5	1633	−8.9

### BSS Treatment Attenuates the Severity of RA in CIA Mice

**FIGURE 5:**
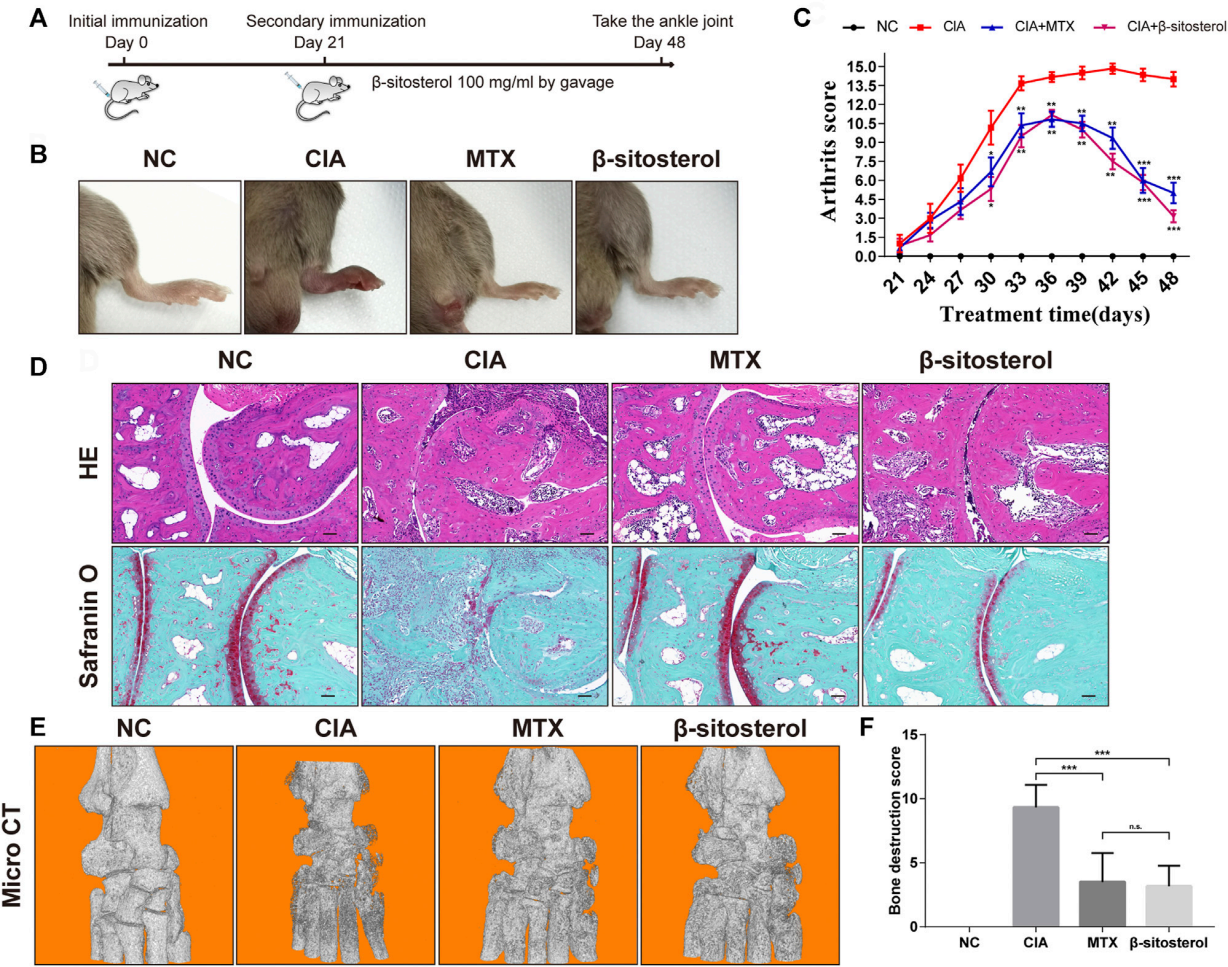
Effects of BSS on experimental arthritis. **(A)** Animal experiment process, **(B)** the mice foot swelling photos, **(C)** Arthritis scores were shown. **(D)** Ankle HE and safranin O-fast green staining in the mice from each group (*n* = 6). **(E)** Micro-CT detection of bone destruction in the ankle joint of each group of mice, and analysis of **(F)** bone destruction scores in each group (*n* = 6). *p < 0.05, **p < 0.01, ***p < 0.001, n.s. = not significant.

The *in vivo* effect of BSS on joint inflammation was evaluated in mice with CIA. As shown in Figure 5A, BSS alleviated the arthritis index score of CIA mice and reduced the degree of ankle swelling **(** Figures 5B,C **)**. Furthermore, compared with the CIA group, HE staining and safranin O green staining revealed that BSS administration reduced the joint inflammation and cartilage erosion **(** Figure 5D **)**. Moreover, micro-CT showed that, compared with the CIA group, BSS (100 mg/kg) ameliorated the bone destruction of ankle in mice **(** Figures 5E,F **)**.

### BSS Treatment Inhibits Synovial Angiogenesis and the VEGFR2 and *p*-VEGFR2 Expression in CIA Mice

**FIGURE 6:**
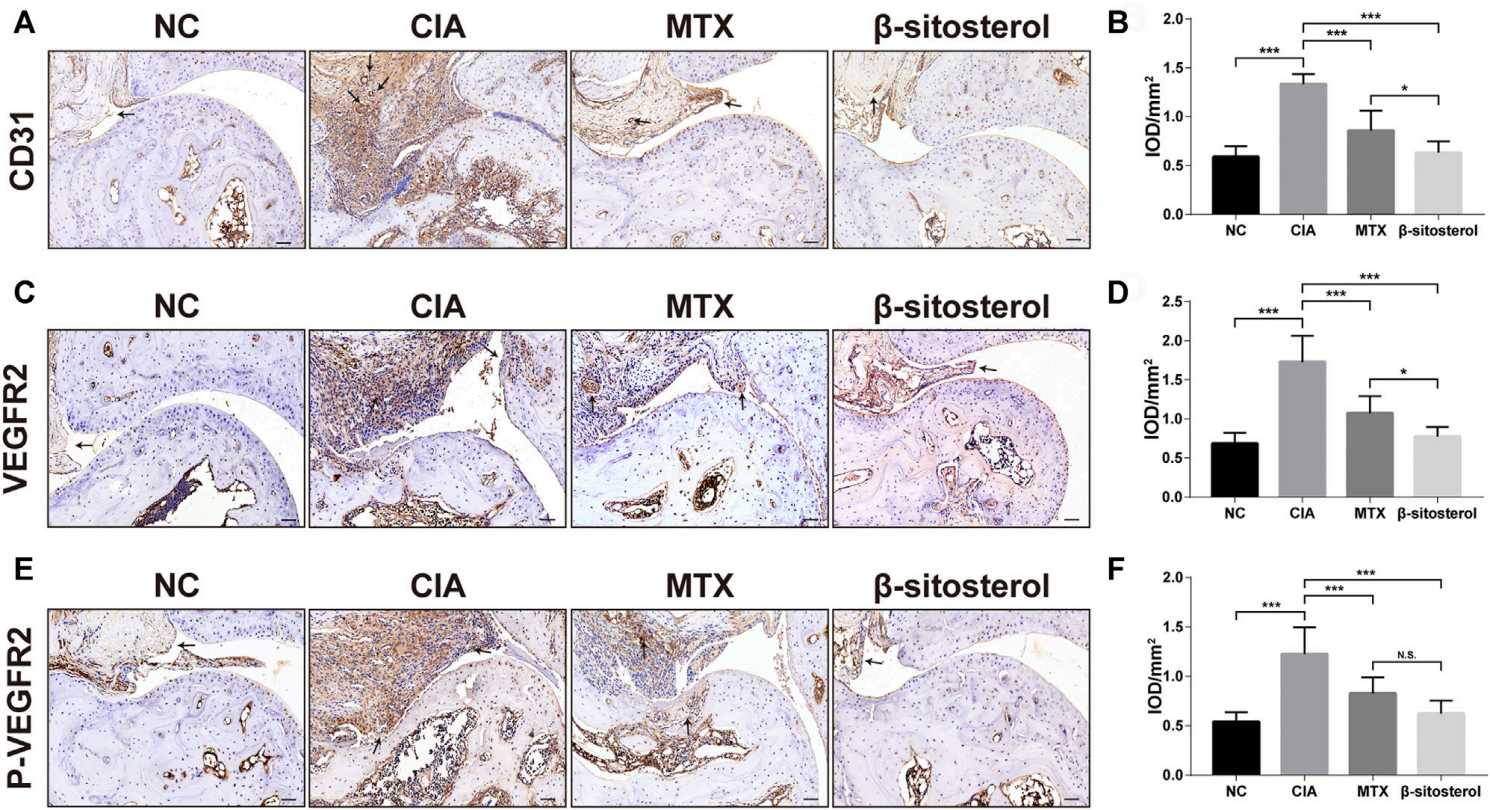
Effect of BSS on immunohistochemistry of CD31, VEGFR2, and P-VEGFR2 on experimental arthritis. **(A)** CD31 immunohistochemical staining of the ankle joints in each group, and the optical density value **(B)** analysis using ImageJ (*n* = 6) (×200, scale bar = 50 μm). **(C)** VEGFR2 immunohistochemical staining of the ankle joints in each group, and the optical density value **(D)** analysis using ImageJ (*n* = 6) (×200, scale bar = 50 μm). **(E)** *p*-VEGFR2 immunohistochemical staining of the ankle joints in each group, and **(F)** the optical density value analysis using ImageJ (*n* = 6) (×200, scale bar = 50 μm). All data are shown as the mean ± SEM. *p < 0.05, **p < 0.01, ***p < 0.001, n.s. = not significant.

We further determined the effects of BSS treatment on CD31, VEGFR2, and *p*-VEGFR2 in ankle of CIA *via* the immunohistochemical examination of synovial angiogenesis. The results showed that BSS (100 mg/kg) significantly inhibited the CD31 expression in the synovial tissues **(** Figures 6A,B **)**. Furthermore, consistent with our *in vitro* results, BSS treatment (100 mg/kg) significantly reduced the expression of VEGFR2 and *p*-VEGFR2 **(** Figures 6C–F **)** positive cells in the synovial tissues of CIA mice.

## Discussion

Synovial angiogenesis is an invasive tumor-like tissue that plays a vital role in the pathological process of RA synovitis and can lead to joint damage and cartilage destruction (Bottini and Firestein, 2013). During the development of synovium angiogenesis, the most important process is the formation of new blood vessels, which is necessary for the expansion, proliferation, and invasion of RA synovium (Fearon et al., 2016). Activation of ECs, including proliferation, migration, adhesion, and tube formation, play a central role in promoting the formation of new blood vessels. This study confirmed that BSS could significantly suppressed the tube formation, proliferation, and migration of HUVECs. At the same time, BSS could also obviously inhibit the formation of ankle and alleviated the degree of joint damage in the CIA mice. Its mechanism was related to the inhibition of VEGF signaling pathway activation.

The different aspects of angiogenesis can be observed in HUVECs capable of simulating the proliferation, migration, adhesion, and lumen formation of ECs. TNF-α and VEGF play a key role in RA and angiogenesis. TNF-α directly affects the migration and proliferation of ECs and the formation of new blood vessels (Leibovich et al., 1987). Meanwhile, studies have shown that TNF-α can affect the level of VEGF in serum of RA patients (Elshabrawy et al., 2015). Therefore, based on the previous studies (Leblond et al., 2020), HUVECs were intervened by TNF-α and VEGF to simulate the *in vivo* environment. We found that BSS significantly inhibited the tube formation of HUVECs and cell proliferation stimulated by VEGF, and prevent the entry of the cell into the S phase. In addition, BSS also restrained migration of HUVECs from the upper chamber to the lower chamber under VEGF stimulation and reduced the formation of stress fibers under TNF-α stimulation. Therefore, BSS may affect the synovial angiogenesis by affecting the proliferation, migration, and tube formation of ECs.

To further evaluate the effect of BSS on synovial angiogenesis and joint destruction *in vivo*, we selected CIA mice to study. CIA is an effective animal model in the study of RA. The pathological changes of CIA mice are highly similar to RA patients, which can simulate the main pathological features of RA (Miyoshi and Liu, 2018). For example, the CIA mice also have pathological changes such as joint involvement, synovial hyperplasia, synovial angiogenesis, bone, and cartilage destruction. The incidence of CIA was 90%–100% at 42–56 days after initial immunization and reached the peak at 40–50 days after initial immunization (Brand et al., 2007). In this study, the joint swelling of mice in the CIA group was obvious, and the success rate of modeling was 100%. The incidence peak reached 51 days after the initial immunization, which was consistent with the description of previous CIA mice studies, and BSS markedly decreased ankle swelling in CIA mice. In addition, HE staining showed the integrity of the articular surface structure of the ankle in the BSS group, but a small amount of inflammatory cell infiltration was observed. Safranin O-fast green staining and micro-CT results showed that compared with the CIA group, the ankle cartilage of mice in the BSS group was more complete, and there was no obvious bone destruction. Overall, CIA mice results confirmed that BSS could significantly improve the joint swelling, protect the integrity of joint structure, and reduce the damage of joint bone and cartilage.

CD31 is a six-domain molecule that mediates the adhesion and the migration of ECs. It is a member of the immunoglobulin superfamily that constitutes the main protein for the connection between ECs. CD31 can be used to show the presence of ECs and help to evaluate the degree of angiogenesis, which expresses on platelets, most white blood cells, and ECs (Liu and Shi, 2012). In the present study, CD31 immunohistochemistry was used to evaluate the synovial angiogenesis in the ankle synovial tissue of CIA mice. The results showed that there was obvious angiogenesis labeled by CD31 in the ankle synovial tissue of CIA mice, and BSS could significantly inhibit the synovial angiogenesis of CIA mice.

Angiogenesis is mediated mainly by VEGF/VEGFR2 signaling, which is considered to be an important target of anti-angiogenic therapies (Karaman et al., 2018). The combination of VEGF with VEGFR2 leads to the phosphorylation of VEGFR2 on multiple tyrosine residues that trigger signaling cascades promoting ECs proliferation, migration, survival, and permeability (Simons et al., 2016). Thus, VEGFR2 activation is a key step in angiogenesis-related course, and blockage of VEGF/VEGFR2 signaling by VEGFR2 inhibitors can suppress angiogenic responses (Roskoski, 2017). In this study, the affinity of BSS and VEGFR2 was predicted by molecular docking. Molecular docking is also able to assess the binding affinity between drugs and targets (Saikia and Bordoloi, 2019), and binding affinity determines bioactivity of drug against the target quantified by the docking score (kcal/mol) (Quiroga and Villarreal, 2016). It was somewhat surprising in our molecular docking results that BSS had a similar binding affinity to VEGFR2 compared with axitinib, a potent inhibitor of VEGFR2 mainly suppressing the phosphorylation of VEGFR2 (Hu-Lowe et al., 2008). According to this finding, it could conceivably be hypothesized that BSS played an analogous role for VEGFR2 block as axitinib. Therefrom, we detected the expression levels of VEGFR2 and *p*-VEGFR2 proteins by Western blot assays, and we indeed found a declining trend of both in HUVECs. On the other hand, we measured their expression in synovial tissues of CIA mice joint using IHC staining that validated our hypothesis. In a word, we confirmed that BSS had an inhibitory effect on the expression and activation of VEGFR2. In addition, previous studies had demonstrated that BSS exerted anti-angiogenesis functions through inhibition of VEGF or inflammatory cytokine expression (Yang et al., 2019). These results added to a growing body of evidence that suggests that BSS probably acted on the VEGF pathway to treat RA.

BSS is considered a safe and potential drug, structurally similar to cholesterol, that inhibits the absorption of cholesterol in the intestines and elevates the levels of enzymatic and non-enzymatic antioxidants, effectively exerting anti-diabetic and lipid-lowering effects (Babu and Jayaraman, 2020). Studies have also shown that BSS has immunomodulatory effects (Fraile et al., 2012), and analgesic and anti-inflammatory effects (Nirmal et al., 2012), which are beneficial for the treatment of RA. In recent years, the effects of BSS in other aspects of RA have also been investigated. In particular, BSS was discovered to reduce the swelling of the ankle and to decrease the levels of collagen-specific antibodies and serum cytokines in CIA mice, by triggering polarization of macrophage to an anti-inflammatory phenotype (Liu et al., 2019). In addition, treatments using the combination of BSS and imperatorin (Guo et al., 2020) and BSS-loaded solid lipid nanoparticles (Zhang et al., 2020) have been reported to ameliorate RA.

In our study, we proved that BSS improved the joint symptoms of CIA mice by affecting synovial angiogenesis. BSS may have a more obvious improvement effect on patients with multiple comorbidities, such as RA with diabetes or RA with hyperlipidemia. In addition, it needs to be pointed out that this study also has shortcomings, and other cell models have not been used for further verification. The effect of BSS on the hypoxia model of HUVECs induced by cobalt oxide and the co-culture model of fibroblast-like synovial cells and HUVECs will be used for in-depth study.

Therefore, we have proved that BSS has a strong restrained effect on synovial angiogenesis *in vivo* and *in vitro*, and alleviated joint swelling and bone destruction in CIA mice. Its mechanism may be related to the suppression of VEGF signaling pathway activation. In a word, BSS might be a potential candidate drug for treating RA.

## Data Availability

The datasets presented in this study can be found in online repositories. The names of the repository/repositories and accession number(s) can be found in the article/Supplementary Material.

## Abbreviations

BSS, β-sitosterol; CIA, collagen-induced arthritis; ECM, endothelial cell medium; ECL, enhanced chemical luminescent; ECs, endothelial cells; GSEA, gene set enrichment analysis; HUVECs, human umbilical vein endothelial cells; RA, rheumatoid arthritis; VEGF, vascular endothelial growth factor.
