# Supplementary material for: β-Sitosterol Inhibits Rheumatoid Synovial Angiogenesis Through Suppressing VEGF Signaling Pathway
Source: Front Pharmacol. 2022 Feb 28;12:816477. doi: 10.3389/fphar.2021.816477 (PMC8918576; doi:10.3389/fphar.2021.816477)
Supplement: Supplementary file 1 [file DataSheet1.docx]

**Supplementary Figure Legends:**

**Sup Figure 1.** **Standardization and differential gene analysis of HC and RA samples in GSE121894 dataset.**

**(A)**Standardized bar chart and **(B)** Volcano Plot for differential gene expression between HC (n =11) and RA (n =19) group in GSE121894 dataset.

**Sup Figure 2. Standardization and differential gene analysis of HC and RA samples in GSE121894 dataset.**

**(A)** p-AKT and p-smad2 protein levels in HUVECs intervened with β-sitosterol and Axitinib were detected by immunoblotting, and the relative expression levels **(B)** of proteins were corrected by GAPDH. **P<*0.05, ***P<*0.01, ****P<*0.001, n.s.= no significant.
